# Complete genome sequence of *Vibrio* sp. RC2502, a crystalline chitin-degrading bacterium isolated from mangrove tidal-flat sediment

**DOI:** 10.1128/mra.00322-26

**Published:** 2026-05-29

**Authors:** Xuebing Ren, Ying Yu, Guangqiang Wang, Xin Song

**Affiliations:** 1 School of Health Science and Engineering, University of Shanghai for Science and Technology 639388 , Shanghai, China; University of Southern California, Los Angeles, California, USA

**Keywords:** crystalline chitin, Chitinase, genomic analysis

## Abstract

We hereby report the complete genome sequence of *Vibrio* sp. RC2502, a gram-negative bacterium isolated from mangrove tidal flats and capable of directly degrading crystalline chitin. The genome has a size of 3,313,050 bp and exhibited a 45.5% GC content, with 2,923 coding sequences.

## ANNOUNCEMENT

Chitin, the second most abundant natural polysaccharide after cellulose (1), can be hydrolyzed into bioactive N-acetyl chitooligosaccharides (2). However, its highly recalcitrant crystalline structure limits biodegradation (3), and few marine microorganisms can directly utilize it (4). In this study, we isolated a crystalline chitin-degrading bacterium, *Vibrio* sp. RC2502, from mangrove tidal-flat sediment collected in Guangdong Province, China (23.56° N, 117.03° E). To isolate the strain, 1 g of sediment was inoculated into 50 mL of minimal medium supplemented with 10 g/L crystalline chitin as the sole carbon source (5). After 3 days of enrichment at 25°C, *Vibrio* sp. RC2502 was isolated from colloidal chitin agar based on clear-zone formation and purified by three rounds of streaking. Its direct utilization of crystalline chitin was confirmed by robust growth in minimal medium containing 10 g/L crystalline chitin.

Strain RC2502 was cultivated in 2216E liquid medium (Qingdao Hope Bio-Technology Co., Ltd., China) at 25°C for 12 h with shaking at 180 rpm. Genomic DNA was extracted from freshly cultured bacterial isolates using an SDS-based method (6), followed by purification with organic extraction and magnetic beads. Genomic DNA was extracted from a single culture, and the same DNA preparation was used for both Nanopore and Illumina library construction.

For short-read sequencing, libraries were prepared from qualified genomic DNA using the NEBNext Ultra II DNA Library Prep Kit for Illumina (New England Biolabs, USA) after fragmentation with a Covaris instrument and were sequenced on the Illumina NovaSeq platform to generate paired-end 150 bp reads. Raw reads were subjected to quality control and adapter trimming using fastp v0.25.0 (7). For long-read sequencing, libraries were prepared from 2.0 to 2.5 μg of DNA using the Oxford Nanopore ligation sequencing kit (SQK-LSK110) and native barcoding kit (EXP-NBD196). The library was then loaded onto an R9.4 flow cell and sequenced on a PromethION platform (Oxford Nanopore Technologies, UK) for 48–72 h. Raw Nanopore sequencing reads were basecalled using Guppy v1.2.0 with the high-accuracy algorithm to ensure high-quality sequence output (8). Nanopore reads were quality-controlled using NextDenovo v2.0 to filter out low-quality reads prior to genome assembly (9).

**Fig 1:**
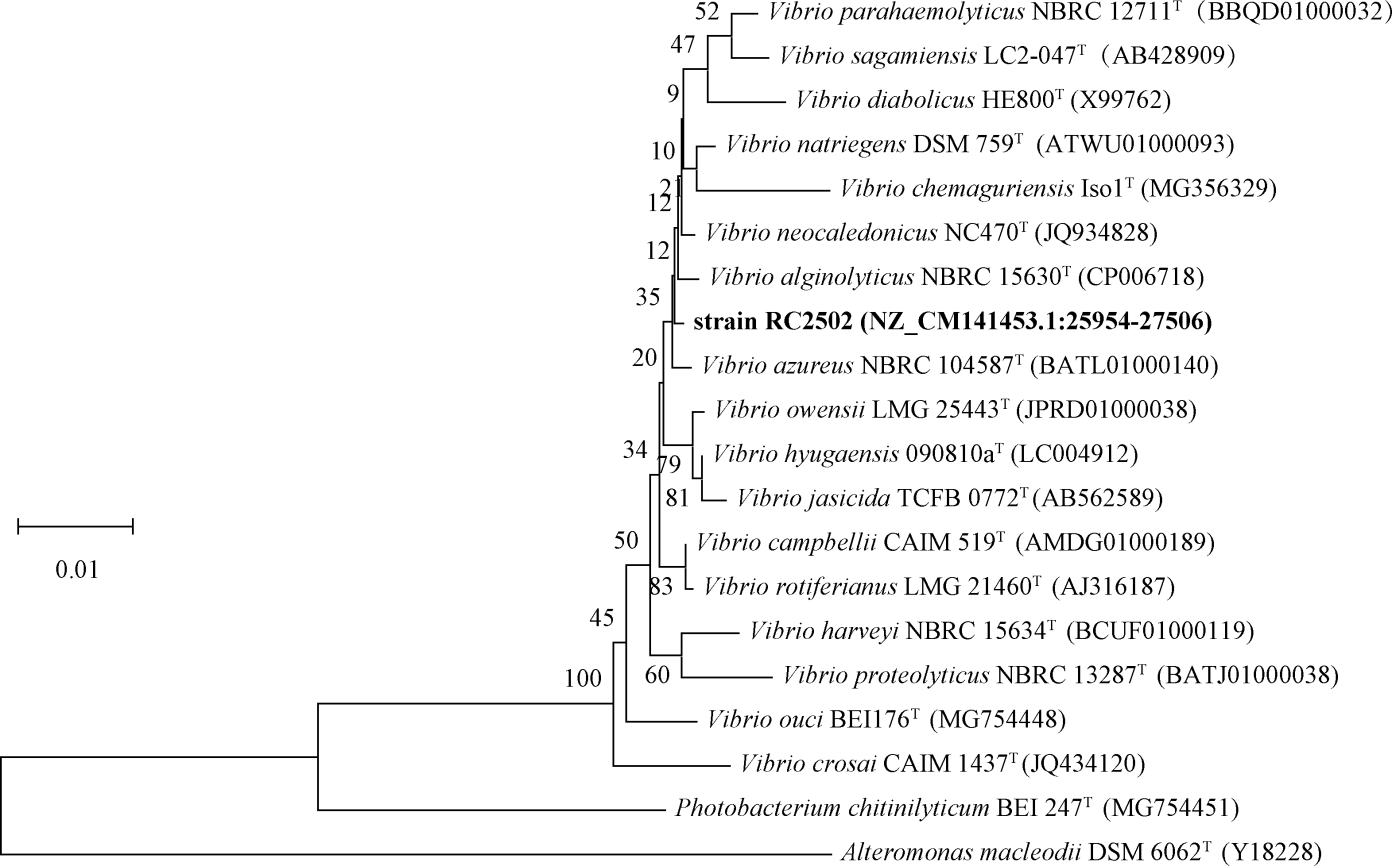
Neighbor-joining phylogenetic tree based on 16S rRNA gene sequences showing the relationships between strain RC2502 and related taxa within the genus *Vibrio*. Bootstrap values (%) based on 1,000 replicates are shown at branch nodes. The scale bar represents 0.01 nucleotide substitutions per site. *Photobacterium chitinilyticum* BEI 247^T^ and *Alteromonas macleodii* DSM 6062^T^ were used as the outgroup.

Hybrid genome assembly was performed using Unicycler v0.4.9 (10), combining Illumina short reads and Nanopore long reads. Genome annotation was carried out using Prokaryotic Genome Annotation Pipeline v6.10 (11). Genome quality was assessed using CheckM v1.2.4 (12), which estimated the genome completeness to be 87.01% with a contamination rate of 0.13%. Based on BLASTN analysis, the 16S rRNA gene sequence of strain RC2502 (from genome, NZ_CM141453.1:25954-27506) showed the highest similarity (99.73%) to *Vibrio alginolyticus* NBRC 15630T (CP006718). Phylogenetic analysis of 16S rRNA gene sequences performed by MEGA X (13) for strain RC2502 and related taxa indicated that strain RC2502 could be affiliated with the genus *Vibrio* (Fig. 1). Default parameters were used for all software unless otherwise specified.

The genome characteristics are summarized in Table 1. In addition, several genes putatively involved in chitin degradation were identified, including ACYJZR_11520, ACYJZR_03005, ACYJZR_03740, and ACYJZR_12245, supporting the capacity of this strain to degrade crystalline chitin.

**TABLE 1:** Genome characteristics of the strain *Vibrio* sp. RC2502

Characteristics	Value
Sequencing and assembly features	
No. of Illumina total reads	5,099,980
No. of nanopore total reads	168,932
Total no. of Illumina bases (bp)	763,098,168
Total no. of nanopore bases (bp)	1,558,768,236
Nanopore N50 (bp)	32,992
Genome coverage (×) (Illumina and Nanopore)	230 and 470
Genome features	
Chromosome size (GC content [%])	3,313,050 (45.5)
Total no. of genes	3,100
No. of CDSs (with protein)	2,923
No. of pseudogenes	21
No. of rRNAs	12, 11, 11 (5S, 16S, 23S)
No. of tRNAs	118

## Data Availability

The complete genome sequence of *Vibrio* sp. RC2502 has been deposited in the GenBank database under accession number JBTVZT000000000, with BioSample number SAMN52939805 and BioProject number PRJNA1354114. The Illumina and Nanopore raw sequencing reads have been deposited in the Sequence Read Archive under accession numbers SRR37718281 and SRR37718280, respectively.
